# Anti-Thyroid Peroxidase and Anti-Thyroglobulin Autoantibodies in the Cerebrospinal Fluid of Patients with Unipolar Depression

**DOI:** 10.3390/jcm9082391

**Published:** 2020-07-27

**Authors:** Rick Dersch, Ludger Tebartz van Elst, Benedikt Hochstuhl, Bernd L. Fiebich, Oliver Stich, Tilman Robinson, Miriam Matysik, Maike Michel, Kimon Runge, Kathrin Nickel, Katharina Domschke, Dominique Endres

**Affiliations:** 1 Clinic of Neurology and Neurophysiology, Medical Center—University of Freiburg, Faculty of Medicine, University of Freiburg, 79106 Freiburg, Germany; rick.dersch@uniklinik-freiburg.de (R.D.); dr.oliver.stich@googlemail.com (O.S.); tilman.robinson@uniklinik-freiburg.de (T.R.); 2 Section for Experimental Neuropsychiatry, Department of Psychiatry and Psychotherapy, Medical Center—University of Freiburg, Faculty of Medicine, University of Freiburg, 79104 Freiburg, Germany; tebartzvanelst@uniklinik-freiburg.de (L.T.v.E.); benedikt_hochstuhl@web.de (B.H.); miriam.matysik@uniklinik-freiburg.de (M.M.); kimon.runge@uniklinik-freiburg.de (K.R.); kathrin.nickel@uniklinik-freiburg.de (K.N.); 3 Department of Psychiatry and Psychotherapy, Medical Center—University of Freiburg, Faculty of Medicine, University of Freiburg, 79104 Freiburg, Germany; bernd.fiebich@uniklinik-freiburg.de (B.L.F.); maike.michel@uniklinik-freiburg.de (M.M.); katharina.domschke@uniklinik-freiburg.de (K.D.); 4 Department of Medicine, Hospital Mutterhaus of the Borromäerinnen, 54290 Trier, Germany; 5 Medical Care Center, Neurology, 78464 Konstanz, Germany; 6 Center for Basics in Neuromodulation, Faculty of Medicine, University of Freiburg, 79106 Freiburg, Germany

**Keywords:** depression, antibody index, autoimmune thyroiditis, thyroid peroxidase, thyroglobulin, Hashimoto encephalopathy

## Abstract

Introduction: The risk of developing depression is increased in patients with autoimmune thyroiditis. Autoimmune Hashimoto thyroiditis is diagnosed using the serum markers anti-thyroid peroxidase (TPO) and anti-thyroglobulin (TG) antibodies. In rare cases, patients with autoimmune thyroiditis can also suffer from the heterogeneous and ill-defined syndrome of Hashimoto encephalopathy. Biomarkers for Hashimoto encephalopathy or for any brain involvement of autoimmune thyroiditis are currently lacking. The aim of the present descriptive study was therefore to determine whether a subgroup of seropositive patients shows intrathecal anti-thyroid antibody synthesis in the cerebrospinal fluid (CSF). Participants and methods: Paired serum and CSF samples from 100 patients with unipolar depression were examined for anti-TPO and anti-TG antibodies using enzyme-linked immunosorbent assays. Antibody-specific indices (ASIs) were calculated for seropositive samples. These ASIs allow the differentiation between the brain-derived fraction of antibodies and antibodies which are passively diffused from the serum. ASIs >1.4 were assessed as positive for brain-derived antibodies. Additionally, for explorative evaluations, a stricter ASI limit of >2 was applied. Results: Anti-TPO antibodies were increased in the serum of 16 patients (16%); increased anti-TPO ASIs (>1.4) were detected in 11 of these patients (69%). Anti-TG antibodies in the serum were detected in three patients (3%), with two of them (67%) showing increased ASIs (>1.4). Overall, the authors found increased anti-thyroid antibodies in 17 of 100 patients (17%), with 13 out of 17 patients showing increased ASIs (76%; range 1.4–4.1). Choosing ASI levels of >2 led to positive findings in six out of 16 patients (38%) with anti-TPO antibodies in their serum but no increase in ASIs in three patients (0%) who were seropositive for anti-TG antibodies. The patients with elevated ASIs (N = 13) were younger than the ASI-negative patients (N = 87; p = 0.009); no differences were noted in the frequency of CSF, electroencephalography, and/or magnetic resonance imaging alterations. Discussion: A subgroup of seropositive patients showed intrathecal synthesis of anti-TPO and, more rarely, of anti-TG antibodies, which might be an indication of central autoimmunity in a subgroup of patients with unipolar depression. The confirmation of elevated ASIs as a biomarker for Hashimoto encephalopathy must await further studies. The relevance of the findings is limited by the study’s retrospective and uncontrolled design.

## 1. Introduction

Patients have an increased risk of developing depression when diagnosed with autoimmune thyroiditis [1]. Autoimmune (Hashimoto’s) thyroiditis is typically detected based on clinical history, symptoms, and a physical examination, as well as an assessment of the thyroid hormone status, the anti-thyroid peroxidase (TPO) and anti-thyroglobulin (TG) antibodies in serum, and the sonography of the thyroid gland [2]. Autoimmune thyroiditis can trigger depressive symptoms via a hypothyroid state that can be treated with L-thyroxin. However, the situation is more complex in the very rarely occurring syndrome of Hashimoto encephalopathy. This syndrome is a heterogeneous, ill-defined disorder associated with depressive, psychotic, and mostly complex neuropsychiatric manifestations that include symptoms such as seizures, confusion, speech disorder, memory impairment, gait disturbances, or stroke-like episodes in patients with autoimmune thyroiditis [3,4,5,6,7]. Affected patients may respond to steroids, leading to the term “steroid-responsive encephalopathy associated with autoimmune thyroiditis” (SREAT) as a synonym [3,4,5,6,7]. According to consensus criteria, Hashimoto encephalopathy is an exclusion diagnosis [8], as no reliable biomarkers have been identified for the presence of Hashimoto encephalopathy [9]. Electroencephalography (EEG), magnetic resonance imaging (MRI), or cerebrospinal fluid (CSF) findings show non-specific findings in patients with Hashimoto encephalopathy [3,4,6,7]. In addition, anti-thyroid antibodies may also be present in more than 10% of healthy individuals (anti-TPO antibodies in 11.3% and anti-TG antibodies in 10.4%) [10], making the antibodies in serum unsuitable for differential diagnoses. Better biomarkers are clearly necessary for the confirmation of Hashimoto encephalopathy [9] or autoimmune brain involvement in patients with depression and Hashimoto thyroiditis.

Measurement of intrathecal anti-thyroid antibody synthesis appears to be a potential biomarker of autoimmune CNS involvement in patients with neurological diseases and schizophrenia [11,12]. Therefore, the rationale of the current study was to investigate if the intrathecal synthesis of anti-thyroid antibodies might also be detected in patients with depressive disorders. For this purpose, serum and CSF samples were examined in 100 depressive patients. Antibody-specific indices (ASIs) were calculated for seropositive samples. ASIs allow the differentiation between the brain-derived fraction of antibodies versus antibodies passively diffused into the CSF from the serum. We hypothesized that a small group of patients with serum anti-thyroid antibodies would show an intrathecal synthesis. As a second step, we analyzed patients with elevated ASIs in more detail and compared them with ASI-negative patients.

## 2. Participants and Methods

This study was part of a larger retrospective research project focusing on the role of CSF biomarkers in patients with schizophreniform and depressive syndromes. The study received approval from the local ethics committee of the University of Freiburg (EK-Fr 609/14). The patients gave their written informed consent before the lumbar puncture.

### 2.1. Patient Group

The authors included 100 consecutive patients since 2006 who had unipolar depression and received a lumbar puncture as part of a clinical diagnostic work-up in the Department of Psychiatry and Psychotherapy of the Medical Center at the University of Freiburg. Lumbar punctures were not routinely offered to all patients with unipolar depression but were reserved for severe cases of endogenous depression or patients with resistance to therapy. Patients with psychoreactive depression were not usually offered lumbar punctures. Only patients who had undergone a complete thyroid hormone status assessment (thyroid-stimulating hormone (TSH), triiodothyronine (T3), and thyroxine (T4)) and who had sufficient residual serum/CSF material for analysis were included. A diagnosis of unipolar depression was established by experienced psychiatrists according to the criteria of the International Statistical Classification of Diseases and Related Health Problems (ICD) - in the 10th revision (ICD-10). The patient group consisted of 25 patients with a first depressive episode (six of these patients suffered additionally from psychotic symptoms) and 75 patients with a recurrent depressive disorder (18 of these patients with psychotic symptoms).

### 2.2. Cerebrospinal Fluid and Antibody Analyses

The lumbar puncture and blood collection were performed at the same time. The diagnostic CSF analysis included the measurement of CSF white blood cell count, CSF protein concentration, CSF/serum albumin quotients, IgG index, and oligoclonal bands. The investigations were carried out in the CSF laboratory of the Clinic of Neurology and Neurophysiology, Medical Center—University of Freiburg. Antibodies for anti-thyroid peroxidase (TPO) and anti-thyroglobulin (TG) were analyzed in paired CSF and serum samples using a commercially available enzyme-linked immunosorbent assay (ELISA; Medipan GmbH, Dahlewitz/Berlin, Germany). The paired serum and CSF samples were measured on the same plate, and the ELISA tests were performed according to the manufacturer’s recommendations, except that the CSF samples were used undiluted as we expected very low CSF titers for the anti-thyroid antibodies. The results were corrected for the different serum and CSF dilutions. Serum samples exceeding the established reference values (anti-TPO antibody levels >50 IU/mL; anti-TG antibody concentrations >100 IU/mL) were controlled in a second ELISA to confirm the first positive findings. When both tests were positive, ASIs were calculated. The methodological procedure was described in more detail in a previous paper [12].

### 2.3. Calculation of Specific Antibody Indices

ASIs were only calculated in patients with elevated serum anti-TPO and/or anti-TG antibodies. For the ASI calculation, the specific anti-TPO/TG IgG antibody concentrations and the total serum/CSF IgG antibody levels were analyzed while considering the blood–brain barrier (BBB) function (i.e., albumin quotients) [13,14]. More precisely, the specific ASI was defined as the ratio between the CSF/serum quotients for the anti-TPO/TG antibodies (Q_spec_) and the total CSF/serum IgG quotient (Q_IgG_) with respect to CSF and serum dilution (ASI = Q_spec_/Q_IgG_). In patients with increased polyspecific intrathecal total IgG synthesis, the use of Q_IgG_ led to an overestimation of the disturbance in the BBB function, resulting in an underestimation of the ASI. In this case, the Q_IgG_ was compared to the CSF/serum albumin quotient (Q_Alb_). The non-linear relationship between both parameters (Q_IgG_ and Q_Alb_) is illustrated in the “Reibergrams” by a hyperbolic curve (i.e., Q_Lim_) to differentiate between intrathecally synthesized IgG and blood-derived IgG. Polyspecific intrathecal IgG synthesis occurs if Q_IgG_ > Q_Lim_. When this occurred, a corrected ASI with CSF/serum Q_Alb_ was calculated using Q_Lim_ = 0.93 × √(Q_Alb_^2^ + 6 × 10^–6^ − 1.7 × 10^−3^) (instead of Q_IgG_). In such cases, the ASI was defined as Q_spec_/Q_Lim_ [13,14]. Normal values are considered to be values between 0.7 and 1.4 [13,14]. A value >1.4 was regarded as an increase and thus as a sign of brain-derived/intrathecal antibody synthesis [12,14]. A further, stricter limit value of >2 was used for explorative analyses [15].

### 2.4. Available Datasets

Thyroid hormone levels and CSF findings were available from all 100 patients. EEGs, however, were only available from 98 patients and MRI results from 87 patients.

### 2.5. Statistical Analysis

The statistical calculations were performed with the Statistical Package for the Social Sciences, version 24 (IBM Corp., Armonk, NY, USA). The results are mainly presented in a descriptive manner. Group differences for dimensional variables were compared using two-sided independent sample t-tests (e.g., for age comparison between ASI-positive and ASI-negative patients). Categorical variables were compared using Chi^2^ tests (e.g., for the analysis of sex ratio between ASI-positive and ASI-negative patients). A significant age difference existed between patients with and without elevated ASIs; therefore, categorical variables with possible age influence (e.g., the number of overall MRI/CSF pathologies) were analyzed using binomial logistic regression. All *p*-values of < 0.05 were considered statistically significant. Due to the exploratory approach, no correction was made for multiple testing.

## 3. Results

### 3.1. Demographic Data

The average age of the 100 enrolled patients (female: N = 55; male: N = 45) was 51.95 ± 19.05 (range 18–85 years).

### 3.2. Thyroid Hormones and Anti-Thyroid Antibody Findings

The mean values of the TSH levels (ref. 0.27–4.20 µU/mL) were 2.06 ± 1.56. Thus, 85 patients (85%) showed normal values, eight patients (8%) showed increased values, and seven patients (7%) showed decreased values. Five of the seven patients (71%) with decreased TSH received substitution treatment with L-thyroxine. The T3 levels (ref. 3.4–6.8 pmol/L) were 4.54 ± 1.01; 92 patients showed normal values (92%), one patient (1%) showed increased values, and seven patients (7%) showed decreased values. The T4 concentrations (ref. 10.6–22.7 pmol/L) were 15.44 ± 4.29 on average; 95 patients (95%) had normal values, two patients (2%) had increased values, and three patients (3%) had decreased values. The anti-thyroid antibody findings are presented in Table 1. Anti-TPO antibodies were increased in the sera of 16 patients (16%), and 11 of these 16 patients (69%) showed increased ASIs. Anti-TG antibodies in serum were identified in three patients (3%), and two of these three patients (67%) displayed increased ASIs. Overall, anti-thyroid antibodies were—in a partially overlapping manner—increased in 17 of 100 patients (17%), and 13 of these 17 patients (76%) showed increased ASIs. 

Choosing a stricter ASI level of >2 led to the detection of increased ASIs in six of the 16 patients (38%) with anti-TPO antibodies in serum, but none of the three seropositive patients for anti-TG antibodies (0%) had increased ASIs. Overall, six of the 17 seropositive patients (35%) had ASIs >2.

### 3.3. Patients with Increased Antibody-Specific Indices

The group of ASI-positive patients (using the reference of >1.4) included more women (9/13 or 69%) than men. Most patients suffered from recurrent depression (11/13; 85%). Thyroid hormone status was normal with or without L-thyroxine substitution in ten of the 13 patients (77%) with increased ASIs. The EEG was altered in four of these 13 patients (31%), and all four patients revealed EEG slowing. The MRI revealed changes in seven of 12 (58%) patients; the changes were mostly non-specific white matter lesions (in 25%). CSF abnormalities were found in eight of 13 patients (62%), mostly as increased protein concentration (in 46%). 

The combined EEG, MRI, and CSF analysis revealed three abnormal signals in four patients (31%), two abnormalities in two patients (15%), one in five patients (38%), and none in two (15%) of the 13 patients (Table 2).

The choice of a stricter ASI level of >2 led to the detection of alterations in the EEG in 17%, in the MRI in 33%, and in the CSF in 50%. Three abnormalities (in MRI, EEG, and CSF) were detected in 17% of the patients with ASIs >2, one in 50%, and no pathology in 33%. No patients showed two changes.

### 3.4. Comparison between Patients with and without Increased Antibody-Specific Indices

Patients with increased ASIs were younger than patients without increased ASIs (39.23 ± 20.60 years versus 53.85 ± 18.17 years; *p* = 0.009). No relevant sex differences were noted in patients with and without increased ASIs (*p* = 0.269). The comparison of EEG, CSF, and MRI overall alterations revealed no significant differences between patients with (N = 13) and without (N = 87) increased ASIs. Table 3 provides an overview of the CSF, EEG, and MRI alterations in the two groups. A total of 24 patients (24%) had unipolar depression with psychotic symptoms, including four patients with elevated ASIs (31% of all patients with elevated ASIs) and 20 of the 87 patients without elevated ASIs (23%). The more prevalent occurrence of additional delusional symptoms in patients with elevated ASIs was not statistically significant (Chi^2^ = 0.375, *p* = 0.540).

## 4. Discussion

The main findings in this study were slightly elevated ASIs (>1.4) for anti-thyroid antibodies in 13% of all patients and clearly elevated ASIs (>2) in 6% of the investigated depressive patients. The ASI-positive patients were younger on average, but, contrary to our expectations, they showed no differences in EEG, MRI, and CSF diagnostic findings when compared with the ASI-negative patients.

Previous studies of patients with neurological disorders and suspected Hashimoto encephalopathy revealed highly positive ASIs (from 5.8–314.5), whereas control patients had no increased ASIs [11]. A subgroup of 65% of the seropositive patients with schizophreniform disorders also showed increased ASIs (>1.4), but the ASI values were less strongly increased in this group (from 1.54–8.16) [12]. The prevalence of ASIs in patients with depressive disorders is in line with earlier results reported in patients with schizophreniform syndromes (ASI detection in 67% vs. 65% of seropositive patients) [12]; however, the ASI maximum values were lower in patients with unipolar depression (range 1.42–4.10). The current and earlier findings suggest that increased ASIs are a transdiagnostic phenomenon, whereas increased ASIs seem to be appreciably higher and more common in neurological patients with Hashimoto encephalopathy [11] than in psychiatric patients.

The direct pathophysiological significance of thyroid antibodies for neuropsychiatric disorders is still unclear. A previous study showed that the anti-TPO antibodies bind to cerebellar astrocytes [16]. Other studies suggested that the smooth muscle cells of cerebral vessels could be target structures for anti-TG antibodies [17,18]. However, the replication of these results is still pending, and their relevance regarding the pathophysiology of depressive syndromes is also unclear. Therefore, most authors consider the anti-thyroid antibodies to be merely an epiphenomenon indicating an autoimmune predisposition [7]. The occurrence of antibodies in healthy individuals [10] and the independence of serum levels from the expression of clinical symptoms in patients with Hashimoto encephalopathy also speaks against a direct pathophysiological significance [4,6,7,19].

The clinical relevance of the present findings might be that the ASIs represent an additional biomarker for a possible autoimmune depression or of Hashimoto encephalopathy. A recently published study has shown that the current criteria for Hashimoto encephalopathy do not predict responsiveness to anti-inflammatory drugs [9], indicating a need for better biomarkers in a situation in which the MRI, EEG, and CSF also provide only non-specific findings [7,8]. After the exclusion of all other causes, such as the presence of all available antineuronal antibodies (including the exclusion of yet unknown antineuronal antibodies with tissue tests), elevated ASIs could indicate central autoimmunity in patients with depression. Further studies treating patients with increased ASIs and other diagnostic alterations (e.g., EEG or CSF pathologies) with anti-inflammatory drugs could investigate whether elevated ASIs have a predictive value for a therapeutic response to anti-inflammatory drugs. Conversely, retrospectively identified groups with confirmed SREAT should be examined for the presence of positive ASIs and compared with non-SREAT patients.

The relevance of the current study is limited by its uncontrolled and retrospective design, a potential selection bias, and the lack of correlation with anti-inflammatory treatment effects. Due to the uncontrolled design, the possible occurrence of elevated ASIs in healthy individuals cannot be ruled out. However, increased ASIs for (antineuronal) antibodies are not expected in control subjects [8,11] and even a positive MRZ reaction with polyspecific increased ASIs for measles, rubella, and varicella zoster is rarely found in controls [20]. The establishment of an adequate control group for this study would require lumbar punctures in mentally healthy adults with Hashimoto thyroiditis, which was not ethically justifiable given the previous unclear study situation. Furthermore, the possibility that ASI-positive depressive patients could have a polyspecific humoral immune response, comparable to a positive MRZ reaction in patients with multiple sclerosis, cannot be ruled out [20]. In addition, a selection bias existed, since only severely affected depressive patients were usually lumbar punctured, and CSF analyses were not performed systematically in all patients with unipolar depression. Accordingly, the results of our study are not representative of all patients with depression. The retrospective design precluded further follow-up of the patients, and none of them were treated with steroids or other anti-inflammatory drugs. Consequently, it remains unclear whether patients with elevated ASIs suffered from SREAT.

## 5. Conclusions

This study provides evidence of an intrathecal synthesis of anti-thyroid antibodies in a subgroup of patients with unipolar depression. Elevated ASIs are interesting biomarkers in neuropsychiatric diseases (compare with their established use, e.g., in Lyme neuroborreliosis) [21], but the role of elevated ASIs for anti-TPO and anti-TG antibodies in patients with unipolar depression requires further intensive research.

## Figures and Tables

**Table 1:** Anti-thyroid autoantibody findings in the whole group of patients with depressive disorders.

Autoantibody	Serum Autoantibodies (N = 100)	ASIs with Reference > 1.4 (with Stricter Reference > 2)	Frequency of Increased ASIs with ASI > 1.4 (>2)
Anti-thyroid peroxidase (TPO) antibodies (ref. < 50 IU/mL)	Increased: N = 16	Increased: N = 11 (N = 6)	In the entire cohort: 11% (6%)
	Not increased: N = 84	Not increased: N = 89 (N = 94)	In the sero-positive patients: 69% (38%)
Anti-thyroglobulin (TG) antibodies (ref. < 100 IU/mL)	Increased: N = 3	Increased: N = 2 (N = 0)	In the entire cohort: 2% (0%)
	Not increased: N = 97	Not increased: N = 98 (N = 100)	In the sero-positive patients: 67% (0%)

Abbreviations: ASI = antibody specific index; ref. = reference value.

**Table 2:** Patients with depression and increased antibody specific indices (ASIs).

Pa.	Age, Sex	Syndrome	Serum TPO/ TG	ASI TPO/ TG	Thyroid Hormone Status	EEG	MRI	CSF	Overall Alterations
1	Mid 60s; Female	Recurrent depressive disorder, moderate to severe episode	TPO: 142.22 (↑) TG: 318.87 (↑)	**TPO: 2.76 (↑)** TG: ↔;	TSH: ↑ fT3: ↔ fT4: ↔(L-thyroxine substitution)	Intermittent theta and delta waves frontally em-phasized and partially on the left hemisphere	Stroke-like lesion in the right stem ganglion; minor micro-angiopathic changes	Increased protein concentration: 575 mg/L	ASI-TPO↑ TSH↑ EEG MRI CSF
2	Mid 70s; female	Recurrent depressive disorder, severe episode	TPO: 119.09 (↑) TG: ↔	TPO: 1.78 (↑) TG: -	TSH: ↔ fT3: ↔ fT4: ↔(L-thyroxine substitution)	Normal	Global cerebral atrophy; minor micro-angiopathy; old lacuna in the anterior capsular limb right	Increased protein concentration: 523 mg/L	ASI-TPO ↑ MRI CSF
3	~20 years; female	Recurrent depressive disorder, severe episode with psychotic symptoms	TPO: 67.59 (↑) TG: ↔	TPO: 1.90 (↑) TG: -	TSH: ↔ fT3: ↔ fT4: ↔(L-thyroxine substitution)	Intermittently fronto-temporally emphasized theta-slowing with intermittent frontal delta waves	Vascular variant	Increased protein concentration: 499 mg/L	ASI-TPO ↑ EEG CSF (MRI)
4	Mid 40s; female	Recurrent depressive disorder, severe episode	TPO: 76.33 (↑) TG: ↔	TPO: 1.75 (↑) TG: -	TSH: ↔ fT3: ↔ fT4: ↔(L-thyroxine substitution)	Normal	Multiple supratentorial white matter lesions on both sides	Normal	ASI-TPO ↑ MRI
5	~20 years; female	Recurrent depressive disorder, severe episode with psychotic symptoms	TPO: 50.49 (↑) TG: ↔	**TPO: 4.10 (↑)** TG: -	TSH: ↑ fT3: ↔ fT4: ↔(no L-thyroxine substitution)	Normal	Wide and asymmetri-cally configured ventricle system; hippocampal malrotation on the right	Normal	ASI-TPO ↑ TSH ↑ MRI
6	Mid 60s; female	Recurrent depressive disorder, severe episode with psychotic symptoms	TPO: 130.44 (↑) TG: ↔	TPO: 1.57 (↑) TG: -	TSH: ↔ fT3: ↔ fT4: ↔(no L-thyroxine substitution)	Normal	Internally accentuated brain volume reduction and single white matter lesions	Only one isolated band in the CSF	ASI-TPO ↑ MRI (CSF)
7	~20 years; female	Recurrent depressive disorder, severe episode	TPO: 94.77 (↑) TG: ↔	**TPO: 3.54 (↑)** TG: -	TSH: ↔ fT3:↔ fT4: ↔(L-thyroxine substitution)	Normal	Normal	Increased protein concen-tration: 719 mg/L; albumin quotient: 8.2	ASI-TPO ↑ CSF
8	~ 30 years; male	Recurrent depressive disorder, severe episode	TPO: 110.05 (↑) TG: ↔	**TPO: 2.04 (↑)** TG: -	TSH: ↔ fT3: ↔ fT4: ↔(no L-thyroxine substitution)	Normal	No conspicuous features (already 2.5 years before antibody testing)	Normal	ASI-TPO ↑
9	Mid 50s; female	Recurrent depressive disorder, severe episode with psychotic symptoms	TPO: 83.34 (↑) TG: ↔	TPO: 1.42 (↑) TG: -	TSH: ↓ fT3: ↓ fT4: ↔(L-thyroxine substitution)	Inter-mittent slowing bi-temporally left emphasized	Several white matter alterations; right parietal defect after brain surgery	Oligoclonal intrathecal IgG synthesis	ASI-TPO ↑ TSH + fT3 ↓ EEG MRI CSF
10	~20 years; female	First, severe depressive episode	TPO: 127.11 (↑) TG: ↔	**TPO: 2.57 (↑)** TG: -	TSH: ↔ fT3: ↔ fT4: ↔(L-thyroxine substitution)	Normal	Normal	Normal	ASI-TPO ↑
11	Mid 20s; male	First, moderate depressive episode with somatic syndrome	TPO: 274.16 (↑) TG: 139.78 (↑)	TPO: ↔ TG: 1.57 (↑)	TSH: ↔ fT3: ↔ fT4: ↔(L-thyroxine substitution)	Isolated diffuse dysrhythmic theta waves as well as IRDAs	Normal	Normal	ASI-TG ↑ EEG
12	~ 50 years; male	Recurrent depressive disorder, severe episode	TPO: ↔ TG: 176.51 (↑)	TPO: ↔ TG: 1.45 (↑)	TSH: ↔ fT3: ↔ fT4: ↔(L-thyroxine substitution)	Normal	Not performed (CT showed an arachnoid cyst)	Increased protein concen-tration: 547 mg/L	ASI-TG ↑ CSF
13	~ 20 years; male	Recurrent depressive disorder, moderate episode	TPO: 312.39 (↑) TG: ↔	**TPO: 2.25 (↑)** TG: -	TSH: ↔ fT3: ↔ fT4: ↔(no L-thyroxine substitution)	Normal	Normal	White blood cell count slightly increased (5 cells/µl); slightly increased protein levels: 451 mg/L	ASI-TPO ↑ CSF

ASIs > 2 are marked bold. Reference values: anti-TPO antibodies < 50 IU/mL; anti-TG antibodies < 100 IU/ ml; ASI < 1.4 Abbreviations: Pa. = patient; TPO = thyroid peroxidase; TG = thyroglobulin; ASI = specific antibody index; TSH = thyroid-stimulating hormone; fT3 = free triiodothyronine; fT4 = free thyroxine; EEG = electroencephalography; IRDA = intermittent rhythmic delta activity; MRI = magnetic resonance imaging; CT = computer tomography; CSF = cerebrospinal fluid; IgG = immunoglobulin G; ↔ normal; ↑ increased; ↓ decrease.

**Table 3:** Additional findings in patients with unipolar depression with and without increased antibody-specific indices (ASIs).

Diagnostic Alterations	Patients with Increased ASIs (N = 13)	Patients without Increased ASIs (N = 87)	Statistics
**CSF alterations overall** **(N = 100)**	Yes: 8 (62%)	Yes: 48 (55%)	W = 1.283
	No: 5 (38%)	No: 39 (45%)	*p* = 0.257
**MRI alterations overall (N = 87)**	Yes: 7 (58%; in one case only a vascular variant)	Yes: 58 (77%)	W = 0.028
	No: 5 (42%)	No: 17 (23%)	*p* = 0.868
**EEG alterations overall (N = 98)**	Yes: 4 (31%)	Yes: 22 (26%)	W = 0.766
	No: 9 (69%)	No: 63 (74%)	*p* = 0.381

The analysis of differences in the rate of overall alterations in the diagnostic findings was carried out using a binomial logistic regression analysis with correction for age (which was different between the two groups). Abbreviations: CSF, cerebrospinal fluid; ASI, antibody specific index; MRI, magnetic resonance imaging; EEG, electroencephalography; W, Wald.
